# Biocatalyst Screening with a Twist: Application of Oxygen Sensors Integrated in Microchannels for Screening Whole Cell Biocatalyst Variants

**DOI:** 10.3390/bioengineering5020030

**Published:** 2018-04-09

**Authors:** Ana C. Fernandes, Julia M. Halder, Bettina M. Nestl, Bernhard Hauer, Krist V. Gernaey, Ulrich Krühne

**Affiliations:** 1 Process and Systems Engineering Center (PROSYS), Department of Chemical and Biochemical Engineering, Technical University of Denmark, Building 229, 2800 Kgs. Lyngby, Denmark; ancafe@kt.dtu.dk (A.C.F.); kvg@kt.dtu.dk (K.V.G.); 2 Institute of Biochemistry and Technical Biochemistry, Universitaet Stuttgart, 70569 Stuttgart, Germany; julia.halder@itb.uni-stuttgart.de (J.M.H.); bettina.nestl@itb.uni-stuttgart.de (B.M.N.); bernhard.hauer@itb.uni-stuttgart.de (B.H.)

**Keywords:** whole cell biocatalysis, biocatalyst screening, microfluidics, oxygen sensors, dioxygenases, organic chemistry

## Abstract

Selective oxidative functionalization of molecules is a highly relevant and often demanding reaction in organic chemistry. The use of biocatalysts allows the stereo- and regioselective introduction of oxygen molecules in organic compounds at milder conditions and avoids the use of complex group-protection schemes and toxic compounds usually applied in conventional organic chemistry. The identification of enzymes with the adequate properties for the target reaction and/or substrate requires better and faster screening strategies. In this manuscript, a microchannel with integrated oxygen sensors was applied to the screening of wild-type and site-directed mutated variants of naphthalene dioxygenase (NDO) from *Pseudomonas* sp. NICB 9816-4. The oxygen sensors were used to measure the oxygen consumption rate of several variants during the conversion of styrene to 1-phenylethanediol. The oxygen consumption rate allowed the distinguishing of endogenous respiration of the cell host from the oxygen consumed in the reaction. Furthermore, it was possible to identify the higher activity and different reaction rate of two variants, relative to the wild-type NDO. The meander microchannel with integrated oxygen sensors can therefore be used as a simple and fast screening platform for the selection of dioxygenase mutants, in terms of their ability to convert styrene, and potentially in terms of substrate specificity.

## 1. Introduction

The use of biocatalysts in industry is increasingly relevant, enabling the production of (new) compounds, some through the conversion of non-natural substrates [1]. Biocatalysts allow stereo- and/or regioselectivity at milder conditions (lower temperatures, close to neutral pH and atmospheric pressure). This is achieved in an often simplified process (remove the need for protection of functional groups in some reactions) that uses less toxic substrates than the equivalent (when existent) organic chemistry methods [1]. However, they can represent a significant fraction of the bioprocess operation cost, due to development costs, need for co-factors, loss of productivity, or complex downstream processing emerging from a low enantioselectivity [2].

Nowadays, around 60% of the industrial biocatalysis is performed using whole cell catalysts [3]. Whole cell biocatalysis solves some of the hurdles of using isolated enzymes, such as catalyst stability in the reaction mixture, enzyme coupling in the cascade reaction system and the need for co-factor addition, by encapsulating the target enzymes in a microbial cell [3,4]. The enzymes involved in the reaction can be from the organism itself but in most cases are from a heterologous source and are inserted in an expression host which is easier to grow, better characterized, considered safe and/or already used in industry (e.g., *Escherichia coli*, *Pseudomonas* sp., *Trichoderma reesei*) [1,5]. In whole cell biotransformations the main parameters to be optimized for process implementation are the oxygen supply, substrate and product toxicity, product stability and co-factor recycling [6]. All the above-mentioned parameters need to be fully characterized and optimized in order to achieve a cost-effective and productive bioprocess [2]. This requires the selection and/or tailoring of the biocatalyst and/or expression host to the desired reaction or process, as well as the optimization of the reaction and process operating conditions through appropriate screening strategies.

Biocatalyst screening, especially for new processes or products or when involving non-natural substrates, is a complex task, involving different levels of screening [7]. Characteristics related to the final process strategy, such as (i) the target reaction or product; (ii) the type of product (intermediate compound, precursor or the final compound); (iii) the industry (e.g., inorganic chemistry, agrochemical, pharmaceutical, food); (iv) the required degree of purity or enantioselectivity desired; and (v) type of application (for profit or for bioremediation, for example) need to be considered and tested during the screening phase in order to find the optimal combination. 

Methods for biocatalyst screening and variant selection require proper design in order to maintain the association between the phenotype observed and the genotype measured [8]. The screening of biocatalysts from initially wide collections, requires the development of both high-throughput growth systems (such as the one developed by Doig et al. (2002) using a robotic liquid handling system [9]) and online quantification and/or monitoring systems.

### 1.1. Screening Approach

The majority of traditional screening approaches are performed in vitro, thus requiring extraction of the sample from the reaction mixture, or to stop the reaction to perform the quantification of the reaction components. On the other hand, in vivo screening (when the biotransformation/bioconversion is monitored in the microorganisms) broadens the number of parameters that can be screened and accelerates biocatalyst development and selection. However, in vivo screening strategies present some limitations associated with the host’s transformation efficiency and growth rate, but also the expression of the target enzyme, limitations in substrate uptake and intracellular background [10]. 

Most assays tend to be specific (to a certain compound or property, e.g., enatioselectivity [11]) to adequately screen for the intended characteristic. They involve standard analytical equipment such as mass spectrometry (MS), gas chromatography (GC), high-performance liquid chromatography (HPLC) or nuclear magnetic resonance (NMR), thus also being quite time-consuming if hundreds or more samples need to be analyzed. This is mainly due to extra steps in sample preparation such as extraction of the compounds, removal of organic phase and chromatographic separation. This tends to increase both the overall analysis time per sample [12], as well as the amount of potential operator errors.

Faster screening methods often involve the formation or consumption of a colorimetric, luminescent or fluorescent compound, which intensity is related with the substrate’s affinity or turnover rate [13]. Spectrophotometric tests are quite desirable due to the possibility of parallelization using microtiter plates, but also owing to the often short measurement time and relatively high sensitivity (signal amplification methods can also be applied). However, most target compounds are neither colorimetric nor fluorescent and developing an indirect colorimetric detection method can be difficult and time-consuming [8]. Moreover, these methods, especially colorimetric methods, can have a high incidence of false positive or negative results which lead to lower precision in the quantification of the target compound [12]. Of the different colorimetric, luminescent or fluorescent methods available that allow long-term measurements, (micro) fluorescence-activated cell sorting ((µ)FACS) devices have been used to screen for growth on a new substrate and to select variants based on enantioselectivity by linking cell survival with capacity for catalysis of only one enantiomer (selection approach) [14,15,16]. Cell survival can also be linked to antibiotic resistance [8]. Flow cytometry, on the other hand, allows assessing whole cell biocatalysts viability and electron transport, besides enzyme activity, achieving a very high throughput (10^8^ screened variants per day [8]). 

Another screening method using reporters allows for an indirect measure of the characteristic target, not by the product or substrate of the bioconversion, but by the genetic reporter that encodes that phenotype. This approach is regarded as reaction independent, and the reporter may be colorimetric, fluorescent, bioluminescent or result in conditional survival, cell motility, acidification, or cell display. The activity of the measured reporter is connected to the activity of the target enzyme by interference at either the transcription (e.g., activation of a natural or synthetic transcriptional regulator by binding of the product or substrate), translation (e.g., by binding of the product to a ribozyme or reporter inactivation by the enzyme) or post-translational modification level (e.g., direct modification of the reporter by the enzyme), or even enzyme degradation or solubility (e.g., by fusing green fluorescent protein (GFP) to the enzyme variant, and thus only soluble GFP variants are positively selected). Reporter-based screening using natural transcriptional regulators are usually extremely selective for the target product, having no false positives, but still requires a case-based choice of reporter and approach [10]. 

### 1.2. Screening Technologies

**Figure 1:**
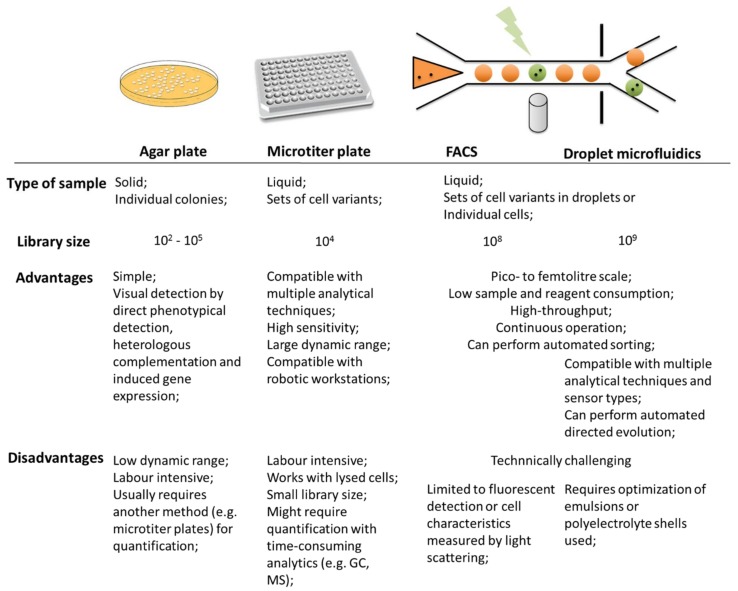
Summary of some of the advantages and disadvantages of the commonly used screening approaches for whole cell biocatalysts. GC: gas chromatography; MS: mass spectrometry.

A summary of the commonly used screening technologies is presented in Figure 1.

Screening in agar plates usually occurs by formation or disappearance of color on the agar surrounding the incubated colonies or by color appearance on the colonies themselves due to bioconversion, providing a straightforward identification of positive colonies. Differences between activity or catalytic rates are, however, not easily achieved with this type of screening and usually up to 10^5^ variants can be analyzed [10]. Screening of 960 variants from cultures grown on a nylon membrane on top of agar plates has been achieved by associating greater intensity of a colored compound with higher producing variants [17]. Coupling of this method with analytical equipment such as HPLC, GC or MS can also be performed. For example, Yan et al. (2017) coupled ambient MS with desorption electrospray ionization (DESI) to achieve a label-free platform for real-time screening of biotransformations in bacterial cultures with imaging MS capable of relating the detected chemical compounds on the surface of the agar plate with their spatial distribution [12]. Metagenomic screening allows the selection of genes with the desired function, by direct phenotypical detection, heterologous complementation, and induced gene expression, usually through isolation in agar plates supplemented with different substrates (e.g., target substrate, certain antibiotics, co-substrates). In this technique, target activity can be further associated with the expression of a reporter gene (e.g., GFP or β-galactosidase) for easier screening [1].

Microtiter plates are the most frequently used screening devices, due to high-throughput and compatibility with a wide range of analytical techniques, from colorimetric approaches to GC, NMR or MS. Microtiter plates are, however, usually limited to libraries of up to 10^4^ variants [10]. UV-Vis and fluorescent microplate readers and fluorescent digital imaging (in which mutants are passed on to a nitrocellulose membrane from the agar plate where they were cultured) [18] can be used to increase throughput during biocatalyst selection. Schwaneberg et al. (1999) developed a high-throughput assay using a robotic workstation and spectral analyzer compatible with 96-well microtiter plates for the analysis of substrate specificity and the activity of mutants of a fatty acid hydroxylating enzyme for both pure enzymes and cell extracts [19]. This method was then further developed to allow measurements directly from the whole cell biocatalysts, eliminating the cell lysis, resuspensions and centrifugation preparation steps, and using a replicator tool (developed by Duetz et al. (2000) [20]) to transfer cells from agar plates to the 96-wells plate. Despite the increase in the number of samples analyzed in parallel (~3000 clones per day) as well as directly from the cell bioconversion, issues of reproducibility between screens and differences in activity relative to the one measured in shake flasks (due to evaporation, a well-known problem in open microtiter plates) were observed [21]. Samorski et al. (2005) developed a system for evaluating induction time and growth differences of whole cell catalysts cultured in 96-well microtiter plates with an integrated optical fiber bundle in a x-y-stage. Measurements of optical density (with light scattering and NADH measurements) and amount of expression of an induced fluorescent protein were performed in all wells, allowing the optimization of the initial cell density and the time of induction, and the online monitoring of product formation in a microtiter plate [22]. Codexis, a company specialized in enzyme engineering, and other enzyme developing companies (e.g., Ingenza (Roslin, UK), Nzomics (Newcastle upon Tyne, UK), InnoSyn (Geleen, The Netherlands), Almac (Craigavon, UK)), provide systems for enzyme screening based on 96-well microtiter plates for ketoreductases, acylases, ene-reductases, transaminases, lyases, dehydrogenases and halohydrin dehalogenases [23]. 

#### Microfluidic Screening Technologies

Microfluidic approaches for the screening of biocatalysts have emerged recently. Microfluidics, due to the ease of flow manipulation and sensor integration, can potentially enable faster measurement and/or the monitoring of a higher number of parameters [24,25,26]. Furthermore, microfluidic systems provide continuous production at lower costs and reagent quantities, as well as offering higher safety and less waste generation. They also allow a better spatial and temporal control of the reactions, and operation at unusual process conditions and catalyst configurations, thus expanding the operation conditions and types of reactions possible [27]. FACS on a chip, for example, provides a better control than regular FACS on the number of cells per droplet (down to single cell), as well as on the number of fluorescent reporters per droplet thus presenting highly quantitative results [8]. Abate et al. (2010) used droplet microfluidics to screen mutants of horseradish peroxidase in yeast cells generated by directed evolution, at rates of thousands per second, being 1000-fold faster than microtiter plate-based robotic screening. In this technique, the reaction was initiated on chip, continued in an incubation section, and the cell containing droplets were dielectrophoretically sorted based on fluorescent intensity using a laser connected to a photomultiplier tube. The microfluidic device was designed to allow reinjection of droplets in the system to allow incubation of low activity mutants for later analysis. The detection limit in this case was of ~3500 molecules in 6 pL or <1 turnover per enzyme [28]. Kintses et al. (2012) has applied a similar strategy, but performing fluorescent detection of enzyme reactions in lysates of single cells of *Escherichia coli* (*E. coli*). The best variants were selected with laser-induced fluorescence in a second device with a dielectrophoretic sorter and broken to isolate their plasmid content and use it for the next evolution cycle. Droplets generated this way, where the target compound can be optically read, can also be screened using FACS. The whole cycle of variants’ screening developed by Kintses et al. (2012) was performed in two days with the analysis of 10^7^ droplets every 3 h [29]. This technique also enables the detection of very small improvements of enzymatic activity [10]. Alternatively, the cells themselves can be used as femtoliter screening vessels, by performing the target reactions inside the cells and detecting the resulting fluorescent product also inside the cells by, for example, FACS. The enzymes can, on the other hand, be displayed on the surface of the cells during the assay by fusion with anchor motifs, being thus more accessible to the substrate (cell surface display technique), but the displayed enzymes may lose activity, and fluorescent substrate or products need to remain bound to the cell surface during measurement. In both approaches, libraries of up to 10^9^ variants can be screened [10]. Droplet-based directed evolution of whole cell catalysts is a very powerful tool to increase screening throughput of engineered biocatalysts, but the use of different sensor technologies (such as near infrared (NIR) [30] or Stroboscopic Epifluorescence Imaging [31], for example) is required to expand its application. Further coupling of such a system with standard analytical equipment such as HPLC, GC or MS would increase its usefulness for biotechnologists and the range of detected compounds. Packer and Liu (2015) provide a good overview of available screening methods for protein selection, as well as a short guide on screening method selection [8].

### 1.3. Screening of Oxidative Biocatalysts

Due to the ubiquitous presence of oxygen and its importance in bioprocesses, screening of biocatalysts in terms of oxygen utilization and/or influence is highly relevant. Of the different enzymes (oxidases, peroxidases and oxygenases) that use oxygen as the electron acceptor during the reaction, the screening of oxygenase variants or mutants is specially complicated. This stems from the fact that the generated products do not cause a change in pH, color or fluorescence, and distinction between regioisomers is often the main objective of the screening program. Also, since most oxygenase substrates and products present poor water solubility, biotransformations are mostly performed in two-liquid phase systems. Furthermore, it is usually applied in whole cell systems and strains that express oxygenases require oxygen not only for the bioconversion but also for endogenous respiration, and so during the biotransformation the oxygen pressure needs to be maintained in order to allow the oxidation reaction to compete with respiration [32]. Among oxygenases, dioxygenases are especially interesting, since they are able to stereo- and regioselectively introduce two oxygen atoms from molecular oxygen in the substrate (e.g., aromatic compounds) [33]. Furthermore, certain types of dioxygenases such as Rieske non-heme iron-dependent oxygenases (ROs), are capable of catalyzing various oxidation reactions (e.g., monohydroxylations, desaturations, oxidative cyclizations) in a variety of substrates, and the range of substrates can be increased (to include non-natural substrates) by changes in the topology of the active site, as demonstrated by Gally et al. (2015) [34].

Screening of dioxygenases is thus usually achieved with more time-consuming and sensitive analytical methods, some of them mentioned above, such as GC or liquid-chromatography (LC) often coupled with MS, or even NMR [32]. These analytical methods can provide a high degree of throughput. However, this throughput is achieved with a lengthy period between variant development, reaction performance and analysis of product concentration and range of compounds. High-throughput screening with robotic microtiter-based devices, as mentioned before, enables the analysis of 100 to 1000 samples of cells per day, but tends to provide hits with low activities and regiospecificity, especially in Gram-positive cells and fungi. Screening can also be performed through enrichment cultures, where the organisms grown on the starting material, might be able to degrade the desired substrate. However, oxidation of the substrate might not occur on the target position [32].

Measurement of the initial oxygen consumption rate in the presence of substrate excess has also been used for characterization of oxygenases, namely dioxygenases [35,36,37]. A study performed by Parales et al. (1999) used oxygen measurements with Clark-type oxygen electrodes to confirm the importance of an aspartate residue for the catalytic activity in Naphthalene dioxygenase (NDO) enzymes, namely in the electron transfer route. When this residue was modified, no oxygen consumption was observed. Furthermore, in this study a stoichiometric consumption of naphthalene and oxygen for wild-type enzymes was observed [38]. In Rachinskiy et al. (2014) monitoring of oxygen levels was used to detect enzyme deactivation as a parameter in a long-term stability enzyme characterization model to predict the process properties of an enzyme in order to aid enzyme screening for industrial applications [39]. Despite the availability of oxygen sensor spots, as well as other formats of oxygen sensors [40,41], for integration in microtiter plates and shake flasks, the oxygen measurement seems to be used mainly as a monitoring or initial characterization parameter, and not as a screening parameter. Oxygen assay studies performed with purified enzyme solutions have indicated that this could be due to the interference of cell respiration in whole cell solutions (where most of the current screening approaches for this type of enzymes are performed). Moreover, the limited application of oxygen measurement as a screening parameter so far might be related to the lower sensitivity of the traditionally used sensors (Clark-type) or the need of working in closed vessels, when working with highly volatile compounds (and so sensors such as syringe or Clark-type cannot be applied). There are more recently developed, highly sensitive and fast oxygen sensors using optical fibers that can contribute to the increase in oxygen monitoring ability in this field and in turn in the knowledge of the role oxygen plays in these biotransformations (e.g., [40,41]). A system capable of monitoring and measuring oxygen levels and its consumption may provide extra input in screening mutants involved in oxygen dependent reactions. Rate of consumption, oxygen availability in the reaction mixture, diffusion limitation of oxygen and/or other substrates, uncoupling and cell density effects are some of the possible biocatalyst characteristics that can be obtained by using more sensitive and integrated oxygen sensors.

Microfluidic systems can enable a good control of diffusion and gradient generation, due to the predominance of laminar flow and mostly diffusion-limited mass and heat transfer achievable at the micro-scale [42], thus providing the perfect environment for studying the influence of oxygen levels on cells. Therefore, in this work, a microfluidic system along with such type of sensors was applied. A meander microchannel with integrated oxygen sensors [43] was here used to monitor and quantify oxygen consumption rate during the bioconversion of styrene to 1-phenylethanediol by wild-type NDO and two NDO variants in *E. coli*. The *E. coli* cells were used as resting cells, since the use of whole cell biocatalysts as resting cells, besides separating growth phase from catalysis, can moreover decrease the competition of cellular reactions such as oxidative phosphorylation with co-factor regeneration [6]. Styrene bioconversion to 1-phenylethanediol was chosen as the reference reaction since styrene has a similar molecular structure to both the native substrate of the chosen enzymes (naphthalene) and the target substrates of the modified enzymes (different alkenes). Hence, styrene was used to compare the different variants in terms of ability to convert this family of substrates. The chosen case study involved the screening of two previously developed dioxygenase variants [44,45] and their comparison with the wild-type NDO. 

In this way, the main goal of this work was to test, as proof-of-concept, whether such a microfluidic system with integrated luminescent oxygen sensors can be used to accelerate the screening of dioxygenase variants, by identifying the earliest reaction time point where a difference in reaction rate can be observed. These reactions are usually performed for 20 h and the mutants evaluated by quantifying product concentration at the end of the reaction by GC [34,44]. By monitoring the oxygen consumption rate at shorter residence times, relative to the conventional approach, during the reaction of different NDO variants, an earlier identification of differences in oxygen consumption may be achieved that may indicate differences in substrate selectivity and/or reaction rate. This may in turn enable a pre-selection of a smaller number of interesting variants to fully test in terms of product identification and quantification with a GC. Thus, the identification of an earlier reaction time where reaction rates are distinct enough to identify a better variant is highly valuable and would furthermore allow a better understanding of the kinetics of the different variants with a potential increase in screening throughput.

## 2. Materials and Methods 

### 2.1. Materials 

All solvents (MTBE, ethanol, 1-octanol), buffer components (potassium phosphate dibasic and monobasic, sodium chloride) and chemicals (styrene, 1-phenylethanediol, indole, IPTG, ampicillin, agarose, yeast extract, tryptone, glycerol, glucose) were obtained from Sigma-Aldrich and Fluka (Steinheim, Germany), Carl Roth GmbH (Karlsruhe, Germany) and Alfa Aesar (Karlsruhe, Germany). *E. coli* JM109/DE3_pDTG141 was obtained by Julia Halder and Prof. Bernhard Hauer (Biocatalysis Group, Institut für Technische Biochemie (ITB), Universität Stuttgart, Stuttgart, Germany) from Prof. Dr. Rebecca Parales (Department of Microbiology and Molecular Genetics, College of Biological Sciences, UC Davis, University of California, Davis, CA, USA) [46].

### 2.2. Heterologous Expression of Naphthalene Dioxygenase (in E. coli)

The general protocol followed to obtain the variants/mutants is described in Gally et al. (2015) [34] and further optimized towards a better reproducibility as presented in Halder et al. (2017) [45]. 

**Figure 2:**
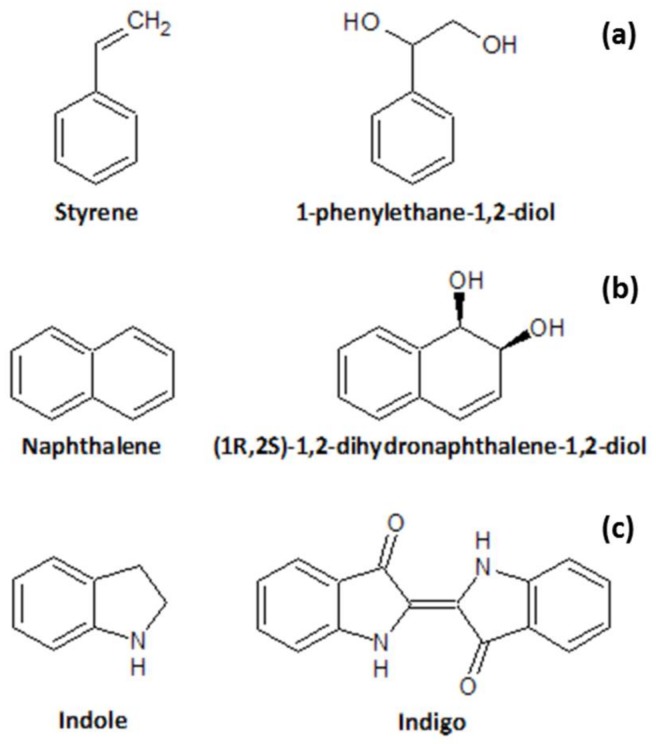
Substrate (left) and corresponding dihydroxylation product (right) for the biotransformation performed (**a**), the natural naphthalene dioxygenase (NDO) substrate (**b**) and the substrate for induction screening, indole (**c**).

To produce induced biomass, *E. coli* JM109 (DE3) cells previously made competent using rubidium chloride, were thawed on ice for 5 min. Then, 1 µL of plasmid DNA for naphthalene dioxygenase (NDO, *Pseudomonas* sp. NCIB 9816-4, pDTG141) or one of the tested mutants was added to the cells and mixed gently by flicking the base of the eppendorf tube and shortly centrifuging. Cell transformation was performed by heat shock by placing the cells in a water bath at 42 °C for 90 s, followed by 2 min on ice. The heat shock treatment was followed by addition of 500 µL of LB medium to the cells and incubation for 1 h at 37 °C and 600 rpm. The competent cells were then plated on selective agar plates containing ampicillin (100 µg/mL) and incubated overnight at 37 °C. To generate the induced cells, one colony from the agar plates was used to inoculate a 2 L shaking flask with 500 mL of TB medium and 500 µL of ampicillin. The flask was incubated at 37 °C and 180 rpm until an optical density (OD_600nm_) of 0.8–1 was obtained. The cells were then induced with 0.1 mM of isopropyl β-d-1-thiogalactopyranoside (IPTG) dissolved in water and incubated at 25 °C for 16 to 18 h. Indole (Figure 2c) was added to the induced cells as a simple screening test in the solid phase in order to check if induction was achieved, since cells successfully induced with NDO or variants produce indigo, turning the media blue [47,48,49]. A representation of the molecular structure of the different substrates used and/or mentioned in the text is presented in Figure 2. Influence of indigo (blue color of the cells) in the optical measurement of oxygen was considered negligible since indigo has a wavelength of 420–440 nm while the laser for excitation of the oxygen sensitive dye emits at 620 nm and the detected excitation light from the sensor dye is 760 nm. After induction, the cells were harvested by centrifuging for 20 min at 6000× *g* and 4 °C in an Avanti J-26XP centrifuge (from Beckman Coulter, Brea, CA, USA) and resuspended in 0.1 M potassium phosphate buffer (pH 7.2) containing 20 mM glucose.

### 2.3. Preparation of Freeze-Dried Cells

To obtain freeze-dried cells, the harvested cells were resuspended in 0.9% sodium chloride (NaCl) solution and centrifuged for 15 min at 4000× *g* and 4 °C. The cells were then spread uniformly in a petri dish and placed inside a freezer at −80 °C for up to 2 h. After freezing the cells were placed inside a freeze drier and freeze-dried overnight under vacuum conditions.

### 2.4. Biotransformation

The cells for the reaction for gas chromatography (GC) validation were prepared by dissolving 0.1 g_cww_/mL and 0.05 g_cww_/mL (cell wet weight) of the freshly prepared resting cells, or 66 mg_cdw_/mL and 33 mg_cdw_/mL (cell dry weight) of the freeze-dried resting cells, in 1 mL of 0.1 M phosphate buffer (pH 7.2) with 20 mM glucose. The cells for the oxygen measurements were also dissolved in 1 mL of 0.1 M phosphate buffer (pH 7.2) with 20 mM glucose, but at lower concentrations (0.005 g_cww_/mL to 0.05 g_cww_/mL). Glucose was added for in situ co-factor regeneration. Immediately, before starting the reaction, styrene (from a stock solution of 100 mM styrene in pure ethanol) was added to the solution to have 1, 1.5 or 2 mM styrene present for the reaction. The reaction was performed in 4 mL vials with a plastic cap (GC and oxygen measurements) or with a plastic cap with a rubber seal (oxygen measurements) in a tabletop orbital MRH11 Heating ThermoMixer (from HLC BioTech, Bovenden, Germany) at 30 °C and 400 rpm (with 3 mm shaking diameter). The rotation chosen to perform the bioconversion was optimized by previously measuring oxygen concentration with reaction time using an oxygen sensor integrated in a syringe tip (Fixed Oxygen Minisensor OXF500PT from Pyro Science, Aachen, Germany) connected to a FireStingO2 Optical Oxygen Meter (from Pyro Science, Aachen, Germany). The rotation which allowed the reaction to be performed with a constant supply of oxygen was the selected one. Two reaction vials were used per residence time, one for GC validation and one for oxygen measurement.

### 2.5. GC Analytical Measurement

The samples for GC analysis were prepared by centrifuging the cells and extracting the supernatant two times with 500 µL of methyl tert-butyl ether (MTBE). The reaction mixture was analyzed at different reaction times by measuring product (1-phenylethanediol) (Figure 2a) concentration in the GC/FID-2010 (from Shimadzu, Kyoto, Japan). A Zebron ZB-1 column (30 m × 0.25 mm × 0.25 µm, from Phenomenex, Torrance, CA, USA) was used, with hydrogen as carrier gas (constant pressure of 50.2 kPa) and the injector and detector at 250 °C and 330 °C, respectively. For the detection, the column oven was set at 70 °C for 2 min, then raised to 120 °C at a rate of 15 °C/min and then raised to 320 °C at a rate of 50 °C/min. The gas chromatography with flame ionization detector (GC-FID) was operated in split mode, using 1 mM of 1-octanol in MTBE as standard. The retention times of all the substances measured are 6.084 min for 1-octanol and 7.729 min for 1-phenylethanediol.

### 2.6. Meander Microfluidic Channel

**Figure 3:**
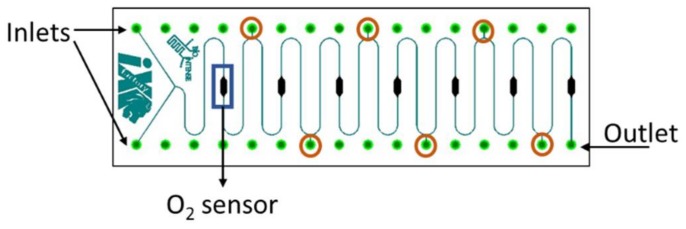
Schematics of the meander microfluidic channel with the main inlets and outlet emphasized. In orange, the five side inlets are highlighted, and in blue one of the seven oxygen sensors is indicated.

The used microfluidic channel is a glass and silicon chip developed and batch-produced by iX-factory (now part of Micronit, Enschede, The Netherlands) in Dortmund, Germany, and has been previously described in Ehgartner et al. (2016) [43]. The microchannel has two main inlets and one outlet, with 6 side inlets/outlets. It has a serpentine shape with 18.5 turns, 0.504 m length and a total volume of 10.08 µL, while the main inlet branched-channels have a volume of 0.44 µL. The microchannel is 200 µm deep and 100 µm wide and presents seven chambers with the same geometry of the sensing areas (3.5 mm length and 1 mm width). The final meander microchannel with integrated oxygen sensors is presented in Figure 3.

### 2.7. Oxygen Measurement Setup

**Figure 4:**
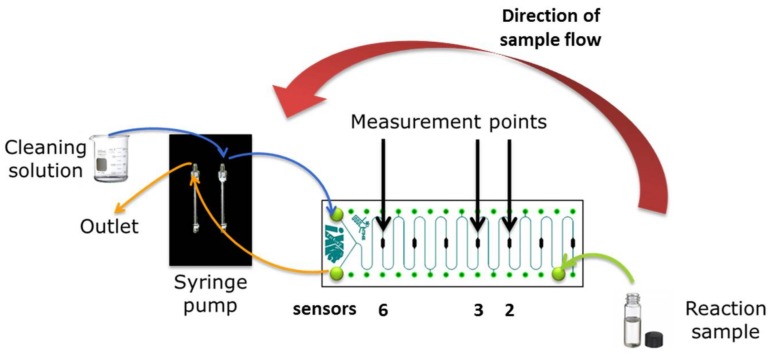
Oxygen measurement setup used for screening variants for styrene biotransformation: pulling approach, with the oxygen sensors position indicated.

The setup used for the measurement of the oxygen consumption rate in the cell samples is presented in Figure 4. The reaction of styrene conversion by the different NDOs was performed in a 4 mL vial and sampled at different reaction times inside the vial. The reaction for each of the residence times considered (1, 3, 5, 15 and 30 min) was performed in a different vial. The sampling and subsequent measurement of oxygen consumption rate was performed in the previously described microchannel with integrated oxygen platinum(II) meso-tetra(4-fluorophenyl) tetrabenzoporphyrin (PtTPTBPF) sensors. More details regarding how the oxygen measurement is performed with this sensors can be found in Ehgartner et al. (2016) [43]. As demonstrated in the setup presented in Figure 4, sampling occurs by pulling a certain volume of the reaction mixture from the vial directly inside the microchannel through the outlet of the meander channel, thus allowing a fast measurement of the oxygen consumption in the sample through single-sampling of the reaction mixture.

The setup presented includes two Cavro^®^ XLP 6000 syringe pumps (from Tecan, Männedorf, Switzerland) with 250 µL syringes controlled with LabVIEW (from National Instruments, Austin, TX, USA). The two syringes were connected to the meander microchannel by polytetrafluoroethylene (PTFE) 1.5875 mm (OD) × 1mm (ID) tubing (S 1810-12) (from Bohlender, Grünsfeld, Germany), using Flangeless polypropylene (PP) fingertight 1.5875 mm (ID) fittings (XP-201) and flangeless ferrules (P200X) (from Upchurch Scientific^®^, Oak Harbor, WA, USA).

**Figure 5:**
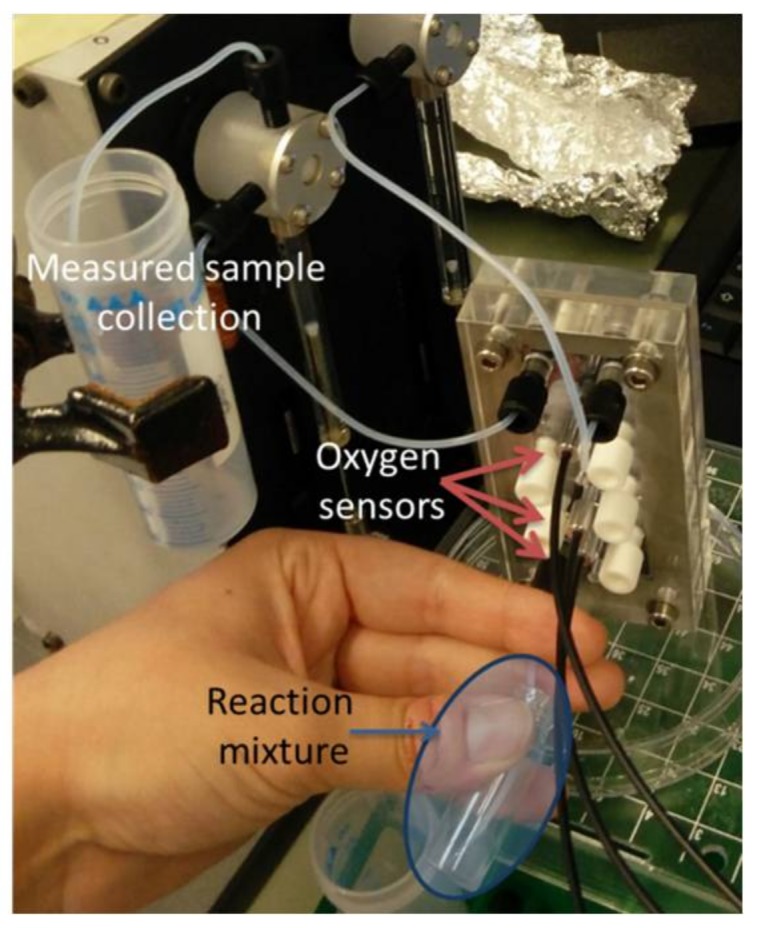
Picture of the pulling sample setup with the meander microchannel in the vertical position.

The cleaning of the microchannel was performed in between samples with ethanol 5% (*v*/*v*) in deionized water followed by solely deionized water. Deionized water was first pulled through the outlet and then the cleaning solutions were pushed through the channel’s second inlet. All the experiments presented in this work were obtained with the meander microfluidic channel placed in an upright position (as presented in Figure 5), to minimize accumulation of cells at the inlet/outlet. 

## 3. Results

This work aims at exploring the feasibility of using a microreactor with integrated oxygen sensors as an alternative screening technology for dioxygenases. An earlier identification of a difference between two variants would allow an acceleration of the first stage of enzyme screening.

To achieve the proposed goal, an evaluation of the following characteristics was performed:

- The microreactor’s ability to distinguish the oxygen consumption due to the reaction from the cells endogenous respiration;
- The microreactor’s ability to distinguish the oxygen consumption rate of different dioxygenase variants during the reaction;
- The microreactor’s ability to differentiate the oxygen consumption rates during the reaction at various substrate concentrations.

**Figure 6:**
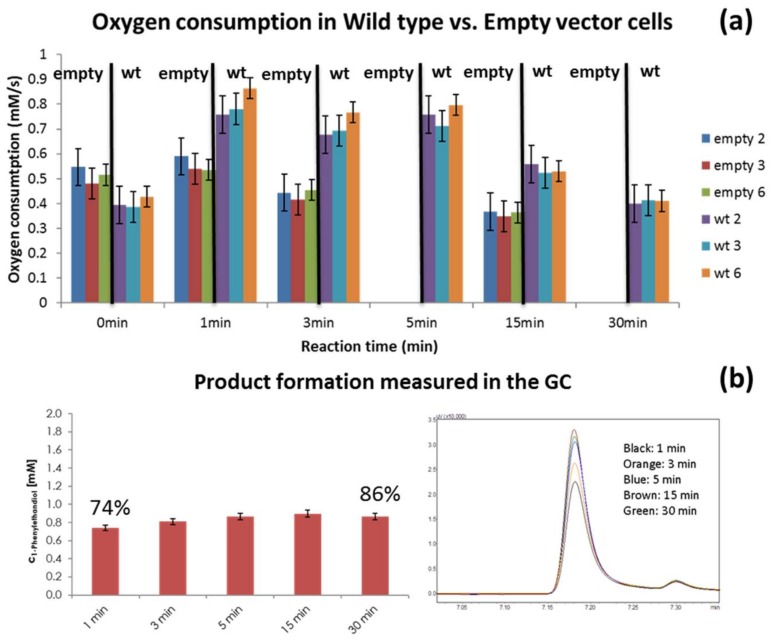
Oxygen measurements of the reaction of empty and wt cells in the presence of 1 mM styrene concentration (**a**); and corresponding GC results for the wt cells of 1-phenylethanediol concentration produced during the reaction and percentage of substrate conversion obtained at 1 and 30 min of residence time (**b**).

**Figure 7:**
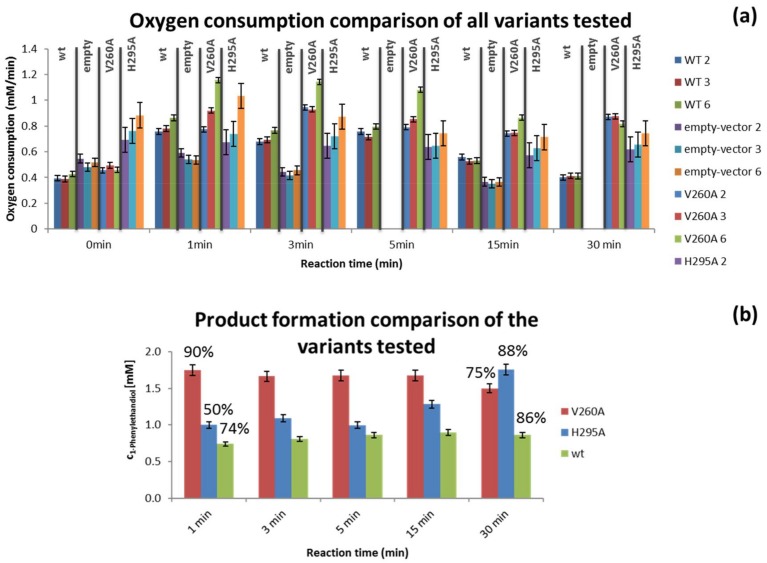
Oxygen consumption rate values for all the cell types tested with the pulling sample approach and higher cell concentration (**a**) and GC results for the two variant containing cells (at 2 mM styrene) and wt cells (at 1 mM) of 1-phenylethanediol concentration produced during the reaction and percentage of substrate conversion obtained at 1 and 30 min of residence time (**b**).

**Figure 8:**
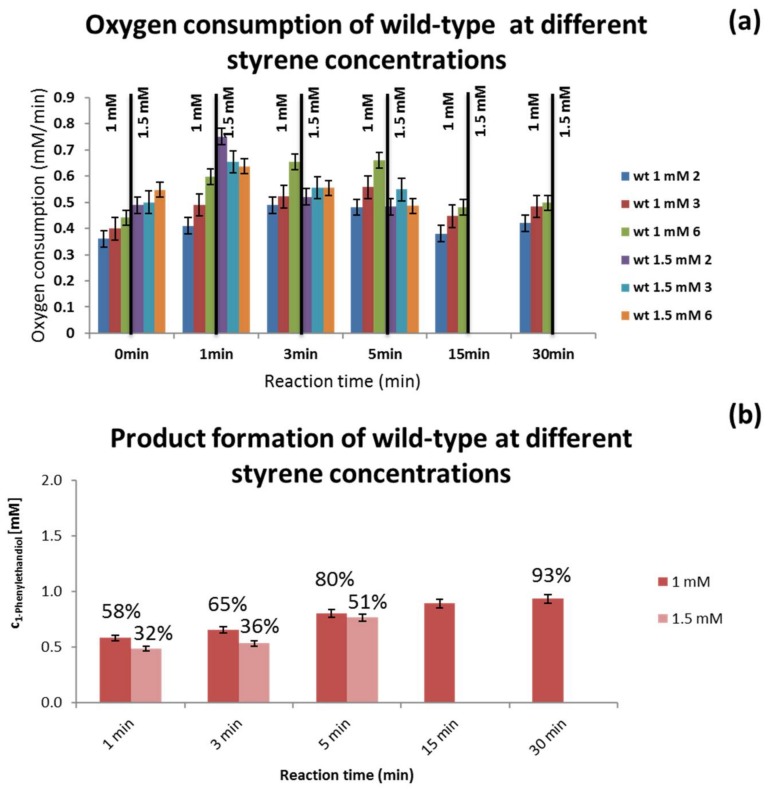
Oxygen (**a**) and GC (**b**) measurements of the reaction wt cells in the presence of two styrene concentrations (1 and 1.5 mM).

**Figure 9:**
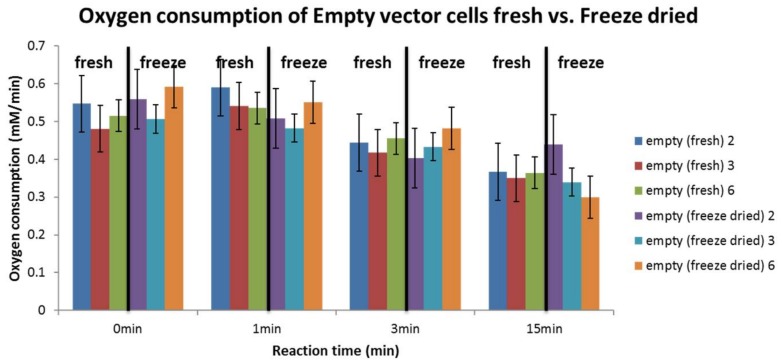
Oxygen consumption rate during the reaction for empty-vector (empty) cells in fresh and freeze-dried preparations at 2 mM styrene.

In the beginning of this study, it was thought that the conversion of styrene required 20 h to reach completion. Furthermore, it was unknown at which point of the reaction a difference in oxygen consumption distinct enough to allow enzyme screening might be observed. As residence times of more than 1 h would be difficult to achieve in the used microchannel (due to its dimension), and it was unknown the number of monitoring (residence time) points that would be required to perform the screening, it was deemed best to perform the reaction in a separate vial and only perform the measurement inside the microchannel. As such, per residence time point, 250 µL of the reaction mixture were sampled from the reaction vial and introduced inside the microchannel at the same flowrate. During the uptake, the oxygen concentration inside the channel was measured. The oxygen consumption rate values were determined from the slope of oxygen concentration during the sampling (oxygen concentration divided by measurement time). The variability of the oxygen measurement was quantified during the reaction with empty-vector cells and is presented through the error bars in Figures 6a, 7a, 8a and 9. In Figures 6b, 7b and 8b the error bars represent the reproducibility of the performed GC measurements.

### 3.1. Reaction vs. Endogenous Respiration

The oxygen consumption rate in the presence of substrate (1 mM of styrene) of empty-vector and wild-type (wt) cells was measured for the selected reaction times: 1, 3, 5, 15 and 30 min, as can be observed in Figure 6a. Less measurements were performed for empty-vector cells since in previous experiments (data not shown) a small change in oxygen consumption value was observed after 3 min, so value at 15 min can be considered as representative of oxygen consumption values of empty-vector cells after 3 min of reaction residence time. Cells containing the wt NDO presented almost 2-fold the oxygen consumption rate of the empty-vector cells in the first 5 min of the reaction. This indicates that there is indeed a higher oxygen consumption in the presence of NDO, which is in principle due to the conversion of styrene by the enzyme. The oxygen consumption rate measured in the three oxygen sensors used (2, 3 and 6) is presented in Figure 6a. Product (1-phenylethanediol) concentration at the different residence times was also measured (Figure 6b) in order to compare with previously obtained results [44,45], and allow a correlation between observed oxygen consumption and substrate conversion. As observed, there is formation of the expected product (1-phenylethanediol) in the presence of the NDO and the high conversion in the initial minutes of the reaction agrees with the observed higher oxygen consumption for the wt cells.

### 3.2. Variant Screening

To evaluate the microreactor’s performance in screening dioxygenase variants, the oxygen consumption rate in the presence of styrene (2 mM) of two NDO-variant containing cells (V260A and H295A) was measured in the meander microfluidic channel. Measurements of product concentration at the different reaction times was also performed in the GC.

Figure 7a presents the comparison of the oxygen consumption values obtained for the variants with the ones previously attained for the empty-vector and wt cells. Variant V260A presents a significantly higher oxygen consumption rate, especially in the first 3 min of the reaction, than wt cells and the other variant. When this is considered together with the GC results it seems to indicate that variant V260A has a faster initial reaction rate, achieving also a higher substrate conversion than wt cells and variant H295A. The latter (H295A), on the other hand, presents a relatively stable oxygen consumption rate during the reaction times monitored, which seems to translate into a slower substrate conversion relative to the other NDOs tested (Figure 7b). The product conversion obtained in the last residence time measured does not differ significantly between the different NDOs, but it decreases in the case of V260A, which may indicate the occurrence of a secondary reaction or further oxidation reactions.

### 3.3. Substrate Concentration Screening

To test the device’s ability to distinguish reaction rate at different substrate concentrations, the oxygen consumption rate and product concentration of wt cells at two different styrene concentrations (1 mM and 1.5 mM) were also measured for the different residence times. No significant difference was observed in terms of oxygen consumption rate, which could be either due to the small difference in substrate concentration or possibly due to mass-transfer limitation of the substrate though the cell wall. The amount of product obtained was also similar for the two styrene concentrations tested, as can be observed in Figure 8b, which seems to indicate mass-transfer limitation constraints.

### 3.4. Influence of Cell Preparation Method

The influence of cell preparation on cell behavior was assessed by measuring the endogenous respiration of freshly prepared empty-vector cells and freeze-dried empty-vector cells in the presence of 2 mM styrene. As shown in Figure 9, no significant difference in the oxygen consumption rate was observed between the two cell preparations, indicating that it is possible to use both fresh and freeze-dried preparations to perform this screening.

## 4. Discussion

The application of the meander channel with integrated oxygen sensors to the measurement of reactions involving whole cells encompassed several challenges. One of the main concerns was the possible clogging of the microchannels due to formation of cell clusters and its effect on fluid flow. *E. coli* cells have dimensions (<2 µm in length) smaller than the microchannels in the meander chip used, and so no significant obstruction of the channels was expected for the relatively diluted solutions used in the experimental work. Another concern involved the cell endogenous respiration rate, which in case of high respiration rates could rapidly consume the oxygen inside the reactor and thus prevent the measurement of the oxygen consumption due to the bioconversion. To address both issues, the cell density was optimized. This was achieved by introducing in the microreactor at a fixed flowrate different dilutions of the wild-type (wt) NDO containing resting cells without substrate present. The resting cells used were *E. coli* JM109 (DE3) cells which were transformed with a pDTG141 plasmid for NDO. The cell dilution considered optimal (0.01 g_cww_/mL) was the one which allowed a good distinction between slow and fast reaction rates, as shown in the Table S1 in Supplementary Materials. The influence of the substrate (styrene) on the oxygen sensors used (which contain polystyrene), was also considered and some tests performed, which are described in the Supplementary Materials.

### 4.1. Measurement Setup

Ideally, the meander microchannel with integrated oxygen sensors would function as an online monitoring system, where the inlet tube connected to the syringe pump would be submerged in the reaction volume, thus allowing sampling from the reaction mixture at given reaction times. This approach, however, was not followed in this study due to several difficulties that could arise from such setup. One of these issues is regarding the high volatility of the substrate (styrene), which could lead to a decrease in the concentration over time, since to sample the reaction mixture, the vial would not be completely sealed. The use of an adequate membrane or a septum could avoid this issue. The other concern relates to the fact that the reaction could be faster than expected and the initial reaction rate missed due to the time taken for sample uptake into the syringe and then introduction in the microreactor.

To avoid these possible issues, a different sampling strategy was applied. The reaction was performed in two identical vials per residence time placed at the same conditions in the tabletop thermomixer. One of the vials was used for measuring the oxygen consumption rate and the other for product quantification in the GC. This enabled a direct correlation between the oxygen measurements and product concentration at the chosen reaction times. Furthermore, by performing the reaction this way, loss of substrate due to its volatility was minimized since the vial was only opened immediately before measurement. To address the possibility of a fast initial reaction rate, sampling uptake was performed through the outlet, instead of pushing the sample through the inlet (see Figure 5). Through this process, by having a short tube at the outlet the sampling time was just a few seconds, instead of close to 1 min, thus allowing monitoring of the reaction at short reaction times (1, 3 and 5 min) and to observe fast initial reaction rates.

### 4.2. Reaction vs. Endogenous Respiration

The chosen sampling strategy allowed measurement of a difference in the oxygen consumption rate between empty and wt cells, as can be observed in Figure 6. Cells containing the wt NDO presented almost 2-fold the oxygen consumption rate of the empty-vector cells. 

It is interesting to observe that 74% of the substrate is converted in the first minute of the reaction. The product concentrations obtained were equivalent to previous lab-scale assays where the reaction had run for 20 h with only end-point GC quantification [45]. After 15 min of bioconversion the oxygen consumption rate decreased, and a stabilization of product concentration is observed, which seems to fit the oxygen consumption rate profile. The reaction was therefore significantly faster than expected and could be monitored with sampling points until 30 min of reaction time was reached. This allows a significant increase in the number of variants and reactions that can be screened per day with the standard method. It is also a good example of the valuable input that microfluidic systems can provide in terms of reaction kinetics in this field. 

### 4.3. Variant Screening

The oxygen consumption rate of the two NDO-variant containing cells (V260A and H295A) was also measured in the meander microchannel. As can be observed in Figure 7a, a difference between the two variants could be observed, as well as differences in oxygen consumption rate relatively to the wt cells and the background (empty cells). One of the variants (V260A) presented a higher oxygen consumption rate, especially in the first 3 min of the reaction. Since we cannot observe what happens in the reaction in the first minute, by being able to observe a higher oxygen consumption, relative to the previous measurements with wt cells, it could indicate that this reaction had a slower initial reaction rate. Thus, the observation of the time at which the oxygen consumption is fastest was possible after the first minute of the reaction. Nonetheless, it might also simply mean that this variant has a higher reaction rate, leading to a faster oxygen consumption rate. As can be observed in Figure 7b, the latter was verified, since a 90% conversion of the substrate was measured in the first minute of the reaction. This variant however, presents a decrease in product concentration at 30 min (Figure 7b) which may indicate further oxidation of the product into another compound or even the production of the other enantiomer of 1-phenylethanediol. 

The other variant (H295A) presented an oxygen consumption rate closer to the wt cells, meaning that the oxygen consumption rate during the bioconversion is very similar to the endogenous respiration for this variant (value of oxygen consumption rate at 0 min in Figure 7a). The observed oxygen consumption rate is also maintained longer above the value for empty cells than for the wt cells. As can be observed in Figure 7, it is also the variant (H295A) that presents the lowest initial conversion with a steady increase during the 30 min of reaction monitored, while the other two cell types maintain approximately the same value after 3 min. Although H295A has a smaller reaction rate than the other measured enzymes, the increased cell respiration might indicate an interference of the heterologous enzyme with the metabolism of the host organism, which is worth of further investigation.

From the presented experiments for empty-vector cells, wt, V260A and H295A, it is possible to conclude that higher consumption rates indicate higher substrate conversion. Moreover, the duration of the oxygen consumption rate above empty-vector cell endogenous respiration levels represents a continuation of substrate conversion, while the decrease in oxygen levels translates in a conservation of product concentration without significant reaction rate.

### 4.4. Substrate Concentration Screening

To understand the applicability of the meander channel as a screening platform, and whether it is possible to distinguish the oxygen consumption rates due to different substrate concentrations, further experiments were performed. The bioconversion with wt cells was performed at two different concentrations of styrene. As demonstrated in Figure 8a, a slightly higher oxygen consumption rate was observed for the higher styrene concentration, but only in the first minute of reaction. The results of substrate conversion obtained from the GC (Figure 8b) indicate that higher substrate concentration results in a lower initial substrate conversion. This can imply some effect of the substrate on the reaction rate. Nevertheless, since the measured product concentrations for the two reactions are quite similar, this might indicate that the reaction rate is limited by substrate diffusion through the cell wall at the concentrations tested. This mass transport limitations are a well-known issue when using whole cell biosystems, which can be improved by permeabilization of the wall with chemical (e.g., adding organic solvents, surfactants, chelating agents or altering the cell wall’s fatty acid content) or physical (e.g., temperature shock, electroporation) methods [50]. Other approaches involve the expression of membrane transporters, to increase influx of substrate to the cell, or the use of cell surface techniques to display the enzymes on the cell membrane, and even temperature-controlled pore-formation through the use of lytic phage proteins [51].

Since no differences between styrene concentration were observed, the distinct oxygen consumption rates observed for the wt cells and the two variants, arise solely from differences in the mutated NDOs.

### 4.5. Influence of Cell Preparation Method

A further test was performed, comparing the measurement performance of freshly prepared cells and freeze-dried cells, since the cell preparation procedure is considerably longer than the time required for the oxygen measurement (Figure 9). The use of freeze-dried cells would enable to perform tests on the same cells for more than one day while guaranteeing their stability and comparative behavior, as well as analyze older cell samples or samples from different sources (e.g., other laboratory facilities) if required. As can be observed in Figure 9, the oxygen consumption rate for wt and empty cells is still very similar for both cell preparations, and so measurements with freeze-dried cells can not only be performed but also compared with the ones made on freshly prepared cells.

### 4.6. Evaluation of Oxygen Measurement in the Microchannel as a Screening Method

To the best of our knowledge, the oxygen consumption rate for dioxygenases in whole cells as presented here has not been previously investigated. Hence, the attained values during the experiments presented here were compared with the only values found for pure dioxygenases (measured with the polarographic method) [35]. The comparison between the values is presented below in Table 1. The literature values (in blue in Table 1) cited in the table were calculated from the values presented in the articles, considering the enzyme concentration (an average value of 250 kDa for the oxygenase component was used for the calculations) used in the respective assays, to have comparable units. The values of this study (in gray in Table 1), are composed by an average of the rates obtained in the first three minutes of the reaction (when the highest rates are measured) in sensor 6 (where the more defined oxygen behavior seems to be observed), calculated without the background value of cell respiration. 

A direct comparison between the values retrieved from the literature and the ones measured in this study is difficult to perform since different enzymes, substrates, substrate concentrations and oxygen measurement techniques were used. There are one or two orders of magnitude of difference in the oxygen uptake between the different catalysts, but it is interesting to notice that the highest uptake rate was obtained for the natural substrate of the enzyme (naphthalene). When comparing biocatalyst performance for the same substrate (styrene), it should be noted that the measurement with whole cells was performed at a substrate concentration that is 10-fold higher relative to the corresponding substrate literature value. If a linear relationship between oxygen concentration rate and substrate concentration is considered, then the rate values obtained for the NDO from *E. coli* JM109/DE3 are half or lower than the one obtained for NDO from *Pseudomonas* sp. NCIB 9816-4. The lower oxygen consumption rate could be related to the already discussed diffusion limitations due to the cell membrane. It should, however, be taken into account that the data presented in this work indicates that the oxygen consumption rates measured for the NDO from *E. coli* JM109/DE3 are diffusion limited and so a linear relationship between oxygen concentration rate and substrate concentration does not occur.

It is also worth mentioning the degree of variability in the data presented here, especially in oxygen consumption rate results which was not possible to quantify appropriately throughout the duration of the experiments. Data variability is most likely related to differences in the preparations of the cell solutions due to small variations in the amount of weighed cells, and thus the number of cells present. However, the variability in oxygen data obtained can also be inherent to the measurement itself, as also described by Jouanneau et al. (2006) with the observed discrepancies in the enzyme activities obtained when the oxygen assay was applied (polarographic assay using Clark-type oxygen sensors) [37].

The presented microfluidic system cannot compete in terms of throughput with most of the screening systems discussed in the introduction. However, it can provide a different type of input (oxygen consumption rate and maybe other oxidative properties) than the above discussed screening platforms. In terms of throughput, the presented system can perform a single measurement every 10 min (including sample uptake and channel cleaning). This means that in a continuous operation, it could perform 47 single measurements in an 8 h working day and 129 single measurements in a 22 h working day (with 2 h for thorough cleaning of the microreactor, tubing and system components). Considering the same reactions performed for this work (around 50 min for each one to be completed), per day we could perform 9 entire reactions in an 8 h working day or 26 entire reactions in a 22 h working day. Thus, to perform measurements every minute of a certain reaction vial, 10 microreactors of this type would have to work in parallel, and such a system is easily achieved with microfluidic units. Furthermore, by working in solution and not in droplet, such a system enables a direct measurement (e.g., side-loop with recirculation) from the reaction vial using a simpler microfluidic arrangement, if a more air-tight setup is assembled. This setup could be achieved by using stainless steel or a less permeable polymer as the tubing material, as well as more appropriate sealed reaction vial caps. The system can also be connected to one of the droplet-based systems discussed in the introduction to achieve a more comprehensive characterization of the mutants during screening.

Moreover, two different screening approaches can be achieved with this platform: variant comparison or substrate comparison. The first approach, which was the one demonstrated in this study, compares the oxygen consumption of different variants relative to the same substrate. A higher rate indicates either higher variant activity or higher substrate specificity for the target substrate. In the second approach, the same cell type can be tested with different substrates. As mentioned, different rates would correlate with differences in specificity and activity. Furthermore, in this case, endogenous cell respiration of the cell without the target enzyme, or of the mutant at different substrate concentrations could further indicate possible toxicity concerns from the substrates being screened.

It is also relevant to mention that the bioconversion at the reaction conditions and cell densities used has a relatively high conversion rate (50 to 70% depending on initial substrate concentration), which greatly contributed to the measurable oxygen consumption difference between the biotransformation and cell respiration. Since higher cell densities might lead to obstruction issues, there is a limitation on the use of this platform in terms of reaction rate to fast reactions only, which generate oxygen consumption rates higher than the host’s respiration rate. This limitation can be observed in Figure 7 for the H295A variant. Reactions with slower kinetics, which is usually the case for initial dioxygenase variants, might hence be more difficult to detect with this microfluidic platform. Faster reaction rates, on the other hand, can be more easily screened, by increasing the flowrate used during the detection and/or by decreasing the cell density used for the biotransformation or for the measurement.

## 5. Conclusions

The presented results have demonstrated the potential of the meander microchannel with integrated oxygen sensors to function as a biocatalyst screening platform for oxygen-dependent whole cell biocatalysts. It was possible to measure the endogenous respiration rate of the *E. coli* cells used and distinguish respiration rate from oxygen consumption due to styrene oxidation by wild-type NDO. Additionally, the two tested NDO variants showed different oxygen consumption rates from the ones obtained with the wild-type. Variant V260A presented a higher oxygen consumption rate in the first 3 min of the reaction, corresponding to a faster styrene conversion (90% after 1 min). On the other hand, variant H295A showed a similar oxygen consumption rate compared to the wild-type NDO, which was equally maintained throughout the monitoring time (30 min), unlike the wild-type NDO, whose consumption rate decreased close to endogenous respiration levels after 15 min. H295A variant, generated however a smaller conversion rate with a steady increase during the reaction time, indicating a slower reaction rate than the other variants tested. Furthermore, this variant (H295A) allowed highlighting of a potential limitation in terms of reaction rates measurable by the microfluidic platform, identifying the reaction rate below which the microfluidic platform is unable to distinguish oxygen consumption due to the biotransformation from the endogenous respiration. The oxygen consumption rates measured with the meander channel with integrated oxygen sensors appear to be concordant with the values found in the literature.

The presented platform shows a considerable potential as a screening platform for mutants involved in oxidative reactions, especially enzymes with high reaction rates and fast bioconversions. The platform can achieve a throughput (129 single measurements or 26 reactions per day) comparable with the current approach, which can be further improved by adding more microfluidic systems in parallel and automating sampling. The current throughput of the platform is similar to the one obtained with the GC, where 129 reactions could be measured within 21.5 h, without time required for reaction preparation (which would imply a further 6 h).

In the future, towards achieving a full characterization of the presented microfluidic system as a screening platform, it would be interesting to perform experiments with additional variants to detect relevant patterns or tendencies observable by using only the oxygen sensors. It would also be interesting to check whether phenomena such as product overoxidation beyond the desired product [32], substrate inhibition or toxicity, or uncoupling effects can be observed or detected through oxygen consumption rate measurements. Uncoupling effects can lead to an increase in oxygen demand, as well as the production of toxic hydrogen peroxide and a lowered specific activity in the final bioconversion. This effect can occur in the absence of substrate, when the substrate cannot be oxidized or in the presence of compounds that do not properly fit the active site [32]. Furthermore, since it was observed that the styrene conversion occurs much faster than initially thought, in a future iteration of the work presented here, the use of the highly controlled flow and residence time dependencies characteristic of microfluidic systems will be used to further characterize the conversion reaction. In this case, the whole cell catalyst will be introduced through one of the channel inlets, and the substrate (or different substrates) introduced through the other. The introduction of the different components inside the microchannel may allow the collection of initial rate values, and a more detailed understanding of the first minute of the reaction.

## Figures and Tables

**Table 1:** Comparison of oxygen consumption rate/oxygen uptake of the whole cell catalyst (in grey) with values for pure dioxygenases found in literature (in blue) [36,37].

Biocatalyst	Substrate	Amount of Oxygenase Component of NDO	Measurement Setup	Oxygen Uptake (mM·min^−1^)
NDO from *Sphingomonas* CHY-1 [37]	Naphthalene (0.1 mM)	PhnI (0.13 µM)	Clark-type oxygen electrode	14.95
NDO from *Pseudomonas* sp. Strain NCIB 9816-4 [36]	Styrene (0.1 mM)	ISP_NAP_ (25 µg)		0.0700
NDO (pDTG141) wild-type in *E. coli* JM109/DE3)	Styrene (1 mM)	-	Luminescent oxygen sensors	0.3202
NDO variant V260A in *E. coli* JM109/DE3)	Styrene (2 mM)			0.6553
NDO variant H295A in *E. coli* JM109/DE3)	Styrene (2 mM)			0.4560

NDO: Naphthalene dioxygenase.

## Supplementary Materials

The following are available online at http://www.mdpi.com/2306-5354/5/2/30/s1, Table S1: Average oxygen consumption rates for different wt cell concentrations calculated for a flowrate of 1.7 µL/s. The cell concentration used for the experiments is highlighted in green.

## Abbreviations

NDO	Naphthalene dioxygenase
*E. coli*	*Escherichia coli*
GC	Gas chromatography
GC-FID	Gas chromatography with flame ionization detector
GFP	Green fluorescent protein
FACS	Fluorescence-activated cell sorting
MS	Mass spectrometry
HPLC	High-performance liquid chromatography
LC	Liquid-chromatography
DESI	Desorption electrospray ionization
NMR	Nuclear magnetic resonance
NIR	Near infrared
ROs	Rieske non-heme iron-dependent oxygenases
PTFE	Polytetrafluoroethylene
wt	Wild-type cells
empty	Empty-vector cells
pDTG141	The NDO-containing plasmid used in this work
