# Supplementary material for: Biocatalyst Screening with a Twist: Application of Oxygen Sensors Integrated in Microchannels for Screening Whole Cell Biocatalyst Variants
Source: Bioengineering (Basel). 2018 Apr 9;5(2):30. doi: 10.3390/bioengineering5020030 (PMC6027248; doi:10.3390/bioengineering5020030)
Supplement: Supplementary file 1 [file bioengineering-05-00030-s001.pdf]

Supplementary materials

1. Cell density and flowrate optimization

Several dilutions of the wild-type (wt) NDO containing resting cells (*E. coli* JM109 (DE3) cells which were transformed with a pDTG141 plasmid for NDO) without substrate present, were introduced at 1.7 µL s^{-1} (0.085 m s^{-1}, Reynolds number (Re) 12.7) inside the reactor and the oxygen consumption was measured (Table S1). The flowrate was chosen based on previous experiments with the empty-vector (empty) cells (cells without the enzyme of interest), where the flowrates were varied between 0.21 µL s^{-1} (0.0105 m s^{-1}, Re 1.56) and 4.17 µL s^{-1} (0.2085 m s^{-1}, Re 31.1). At higher flowrates, the cells tended to accumulate faster both at the outlet and inside the channel resulting in a rapid oxygen consumption and leakage issues. Thus, lower flowrates were more appropriate to perform the reaction. However, at very low flowrates, the oxygen inside the microchannel was completely consumed, leading to the selection of an average flowrate (1.7 µL s^{-1}) as the optimal flowrate for the experiments.

As shown in Table S1, a considerable dilution of the initial sample (1:5) was required in order to achieve a rate of oxygen consumption appropriate for sample comparison. The initial cell concentration was 0.05 g_{cww}/mL, which was half of the cell density usually used for the reaction at lab scale. Since variants with a higher affinity for the substrate or higher reaction rates could result in a faster oxygen consumption, the target oxygen consumption rate of the wt cells (without substrate) had to allow the distinction between faster and slower rates. The value considered as "good" for the purpose of these experiments was around 0.02 mM/min, as highlighted in the table below. Higher dilutions did not allow a detectable reaction rate (data not shown).

Table S1. Average oxygen consumption rates for different wt cell concentrations calculated for a flowrate of 1.7 µL/s. The cell concentration used for the experiments is highlighted in green.

Cell concentration (g_{cww}/mL)	Dilution of initial sample	Oxygen consumption rate (mM/min)
0.05	1	-0.061
0.025	1:2	-0.051
0.01	1:5	-0.017
0.005	1:10	-0.019

2. Styrene concentration optimization

The concentration of styrene used for the reaction also required consideration. Since the integrated oxygen sensors are made of polystyrene (polymer chains of styrene monomers), high concentrations of styrene could dissolve the polymeric layer and release the trapped dye, thus causing bleaching of the sensors and loss of sensing ability. Furthermore, a high styrene concentration could also lead to a faster reaction rate and thus faster oxygen consumption, impacting the previously determined optimal flowrate to perform the reaction. In order to test the effect of styrene, droplets of 100 mM styrene solution (50 to 100 times more concentrated than in the experiments), were placed on sensor spots, with no significant changes observed in signal detection. However, since the sensor spots were placed in an open environment, a rapid evaporation of styrene was observed and so the true effect of styrene might not have been measured. The lack of alternative testing devices, besides the meander microchannels used for the experiments, impeded further tests of the styrene effect on the oxygen sensors. The concentrations of styrene chosen to perform the reactions were thus based on the ones used for the lab scale bioconversions, 1 and 2 mM of substrate.
